# Innovative Approaches to Brain Cancer: The Use of Magnetic Resonance-guided Focused Ultrasound in Glioma Therapy

**DOI:** 10.3390/cancers16244235

**Published:** 2024-12-19

**Authors:** Aleksandra Ćwiklińska, Dominika Przewodowska, Dariusz Koziorowski, Stanisław Szlufik

**Affiliations:** Department of Neurology, Faculty of Health Sciences, Medical University of Warsaw, 03-242 Warsaw, Poland

**Keywords:** FUS, glioblastoma, glioma, focused ultrasound, targeted therapy

## Abstract

Gliomas are a wide group of common brain tumors, with the most aggressive type being glioblastoma multiforme (GBM), with a 5-year survival rate of less than 5% and a median survival time of approximately 12–14 months. The standard treatment of GBM includes surgical excision, radiotherapy, and chemotherapy with temozolomide (TMZ). However, tumor recurrence and progression are common. Therefore, more effective treatment for GBM should be found. One of the main obstacles to the treatment of GBM and other gliomas is the blood–brain barrier (BBB), which impedes the penetration of antitumor chemotherapeutic agents into glioblastoma cells. Nowadays, one of the most promising novel methods for glioma treatment is Magnetic Resonance-guided Focused Ultrasound (MRgFUS). Low-intensity FUS causes the BBB to open transiently, which allows better drug delivery to the brain tissue. Under magnetic resonance guidance, ultrasound waves can be precisely directed to the tumor area to prevent side effects in healthy tissues. Through the open BBB, we can deliver targeted chemotherapeutics, anti-tumor agents, immunotherapy, and gene therapy directly to gliomas. Other strategies for MRgFUS include radiosensitization, sonodynamic therapy, histotripsy, and thermal ablation. FUS can also be used to monitor the treatment and progression of gliomas using blood-based liquid biopsy. All these methods are still under preclinical or clinical trials and are described in this review to summarize current knowledge and ongoing trials.

## Simple Summary

Gliomas are a group of brain tumors, with glioblastoma multiforme (GBM) being the most aggressive and difficult to treat. GBM has a very poor prognosis, with a 5-year survival rate below 5%. Standard treatment includes surgery, radiotherapy, and chemotherapy using temozolomide (TMZ), but tumor recurrence is common. A major challenge in treating GBM is the blood-brain barrier (BBB), which limits the effectiveness of chemotherapeutic drugs. One promising approach to overcoming this barrier is Magnetic Resonance-guided Focused Ultrasound (MRgFUS). MRgFUS uses low-intensity ultrasound waves to temporarily open the BBB, allowing drugs to reach brain tissue more effectively. The ultrasound can be precisely directed to the tumor, minimizing damage to healthy brain tissue. MRgFUS also enables the delivery of targeted treatments, such as chemotherapies, immunotherapies, gene therapies, and even radiosensitization. Additionally, MRgFUS can be used for other therapies like sonodynamic therapy, histotripsy, and thermal ablation. It also offers potential for monitoring treatment progress through blood-based liquid biopsies. These methods are still being tested in preclinical and clinical trials, but they represent a promising new direction for improving glioma treatment. This review discusses the latest advances and ongoing trials related to MRgFUS in glioma therapy.

## 1. Introduction

Glioblastoma multiforme (GBM) is the most aggressive primary brain tumor and is classified as a World Health Organization (WHO) grade IV astrocytoma. It is characterized by rapid proliferation, extensive infiltration into the surrounding brain tissue, and a poor prognosis, with a median survival of approximately 15 months, despite aggressive treatment [1]. GBM occurs predominantly in adults and presents with a variety of symptoms, including headaches, seizures, and neurological deficits, depending on its location in the brain [2].

The treatment of GBM typically involves a multimodal approach that includes surgical resection, radiation therapy, and chemotherapy. The standard initial treatment is the maximally safe resection of the tumor, followed by adjuvant radiotherapy combined with the chemotherapeutic agent temozolomide (TMZ) [1]. This regimen aims to reduce the tumor burden and delay recurrence. Despite these interventions, GBM often recurs, necessitating further treatments, such as additional surgery, re-irradiation, or experimental therapies, including targeted therapies and immunotherapy [3,4]. Recent advancements in understanding the molecular landscape of GBM have led to the exploration of personalized medicine approaches, although challenges remain in effectively targeting tumor heterogeneity [5].

## 2. Magnetic Resonance-guided Focused Ultrasound (MRgFUS) Overview

### 2.1. Mechanism of Focused Ultrasound (FUS)

**Figure 1:**
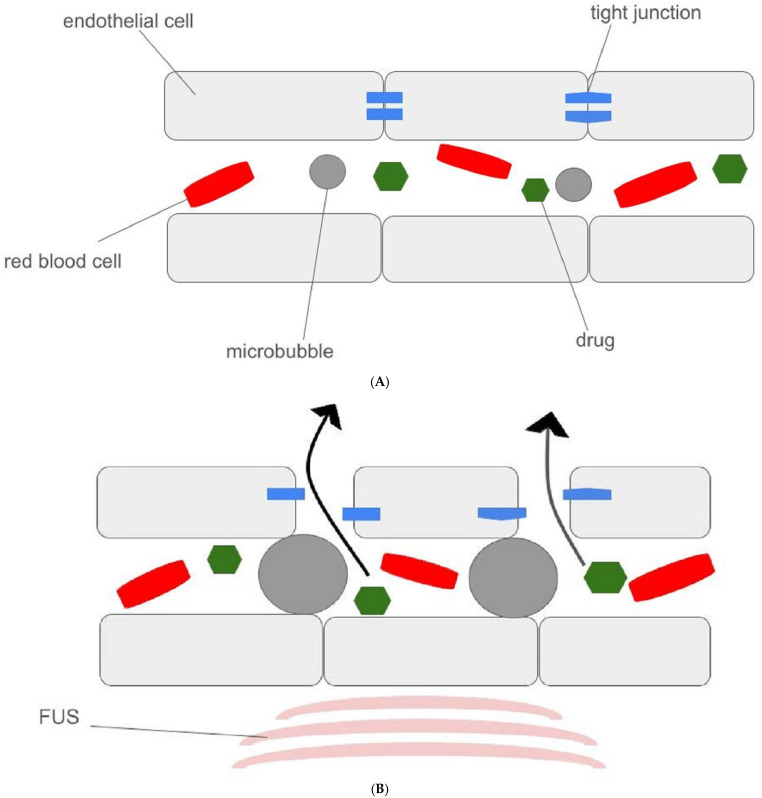
The mechanism of blood–brain barrier (BBB) opening by focused ultrasound: (**A**)—the normal blood flow through brain vessels; (**B**)—the impact of focused ultrasound on BBB, resulting in the drug crossing into brain parenchyma due to acoustic radiation forces provided by microbubbles.

The idea of using microbubbles in the animal model of an ultrasound-treated area came from Hynynen et al. [6]. It was postulated that the blood–brain barrier (BBB) could be easily crossed due to acoustic radiation forces providing microbubble (MB) cavitation and both their direct and indirect interactions with blood vessel walls [7]. Due to the vessel stretching, the temporary opening of intercellular junctions or altering the expression of proteins forming this mechanical barrier can be provided [8,9]. The mechanical pump effect—pushing fluids into the cerebral parenchyma, resulting from arterial undulation—is also mentioned as a factor that enhances MBs’ therapeutic effect [10] [Figure 1].

A microbubble is 1–2 µm in diameter, composed of a gas-core and a shell made of proteins and lipids [11,12,13,14]. Their diameter should be lower than 10 µm to provide their safe removal through pulmonary capillaries and follicles [15]. The average time of their presence in the bloodstream is estimated as 10–15 min [16,17,18]. In smaller vessels (10–20 µm diameter), without a smooth muscle layer in their structure, MBs can contact the vascular wall more closely and for a longer time, providing greater efficacy of applied therapy [16,19,20,21,22,23,24,25,26]. The inner gas can easily disperse out of the bubbles, so it is relevant to maintain the stable composition of the bubbles’ outer membrane and provide their regular circulation in the bloodstream after injection [11]. As a result of quick gaseous instability and rapid dissolution in the blood environment, typical microbubbles are additionally equipped in the lipid or albumin sheath, protecting their air-resembling core [27]. Interestingly, although the inner MB layer made of clear oxygen is suspected to deplete MBs’ structure faster than other inserted gases, environmental oxygenation is described as a common method to increase tumor radiation sensitivity [28,29,30].

Over time, this technique has been modified: acoustic waves have been transformed into high-energy pulses, leaving the track of the beams uninjured. The method of focused ultrasounds exhibits multi-faceted effects, resulting from the action of both mechanical and thermal effects.

The level of acoustic energy used during ultrasound investigation provides different microbubble activities, called stable or inertial cavitation, revealing distinctive results [31]. While stable cavitation allows for increased permeability and endocytosis across its structure, inertial cavitation leads to rapid changes and displacements of much larger structures.

Low acoustic emission provides chemical rectified diffusion, resulting in the expansion of bubble size and its minimal oscillation. A bubble emits a harmonic signal, and its nearest environment starts medium-eddying called “microstreaming”, which leads to physical stress acting against endothelial cells and results in tissue disruption [32,33,34,35]. Due to the activation of ion channels (sensitive to mechanical stimuli), the BBB’s permeability increases [7,36].

On the other hand, the increased level of acoustic energy used during investigation and the higher pressure amplitude can lead to the rapid growth of the bubble and its collapse [37]. The quick reaction is accompanied by great quantities of reactive oxygen species, increased local temperature, high-pressure exertion, and jet formation [38,39,40,41,42,43,44]. Asymmetric fragments of MBs from cavitation can mechanically interrupt the cellular membrane, called jetting. Increased MB oscillation amplitude can lead to sonoporation—membrane pore formation [16]. Newly created orifices’ and pores’ ability to reseal concomitantly with the rapid calcium influx can result in temporary BBB attenuation or even cell death [45,46,47]. Influence on actin and tubular networks (cytoskeletal elements) and endocytosis promotion are also known as activities strictly linked with sonoporation [15,48,49,50,51,52]. Increased aquaporin-4 expression due to the greater ultrasound pressure could also provide an enhanced permeability of the BBB [53]. Tumor-associated microglia and macrophages (TAMs) could remain almost one-third of the GBM structure. Due to the fact that they play a role in the extracellular matrix (ECM) degradation and stimulate angiogenesis, they are believed to enhance GBM progression [54]. Microglia-derived TGF-beta triggers matrix metalloprotein (MMP)-2 expression, providing the degradation of cellular basal membrane and reducing tumor cell restrictions [55].

An abundant amount of energy can result in inflammation-induced glial formation, edema, cerebral hemorrhage, and even cell death [36,56,57,58]. Although sterile inflammation remains a confirmed side effect of FUS-induced MBs’ activity, its presence is strictly dependent on the level of acoustic pressure and dosage of MBs used during the trial. Lower levels of pressure provide safe BBB disruption, whereas increased oscillations lead similarly to the previously mentioned upregulation of NF-KB path-signaling molecules and a wide spread of pro-inflammatory particles, including chemokines (CCL2, 3, 4, 12, 17, CCR5) and receptors for immunoglobulins (FCGR1) [36,59]. Microglial response marked by an increased level of Iba1 (calcium-binding adaptor molecule 1) and astrocytic reaction determined by GFAP elevation remain quick and modest and do not persist permanently [60,61,62,63,64]. It is worth mentioning that the rise in the microglia concentration is combined with the increase in anti-inflammatory A2-reactive astrocyte levels at 48 h after injection, confirming systemic immune response and its promptness to recovery [36,65,66,67,68]. Interestingly, microglial and astrocytic responses co-existing with transcriptional modification activity were observed even two weeks after sonication [14,69]. Regarding the genomic alternations, an increased level of complementary response genes (C4b, C3ar1, C5ar1, C1qa) was also described.

### 2.2. HIFUS and LIFUS

The biological influence of low-frequency ultrasound (LIFUS) on voltage-gated ion channels results in numerous alterations in cellular activity. A single geometrically focused transducer generating waves with almost 330 times lower intensity than HIFUS determines the need for a decreased amount of energy management, contributes mainly to sonoporation, increases the permeability of continuous barriers such as the BBB, and does not remain permanent [70,71]. Neuromodulation can also be established by shifts in potassium channel permeability or as a result of the kinetic energy influence generated by ultrasound on the mechanosensitive membrane and their rearrangement [72]. This can lead to the stimulation or inhibition of cortical electrical activity and can persist for a couple of hours or even days after LIFUS intervention [70,72,73]. The enhancement of excitatory neurons located in the prefrontal cortex and inhibition of the proinflammatory TLR4/NF-KB path led to the attenuation of social avoidance in animals [74,75]. The influence on the neuroplasticity and modification of post-synaptic protein expression provided in the rat depression model underlines the broad potential of LIFUS in numerous diseases [75]. Studies based on animal models of neuropathic pain have revealed that LIFUS also contributes to a decrease in pro-inflammatory cytokines (TNF-α, CNTF, and IL-1 beta) expressed in rat tissues, revealing immunomodulatory properties [76].

The use of higher energy in the HIFUS method is primarily used during treatments aimed at precise removal from the bodily concrete tissues, dependent on the increased wave frequency leading to the generation of an increased amount of absorbed energy. MRgFUS, based on the connection between MRI and FUS, remains the safest and most effective method to localize the lesion and observe the potential effects of this type of therapy. The accuracy of the HIFU ablation method is up to 10 cells (250–300 microns) [70]. The contemporary FUS technique consists of a hemispheric helmet around the head equipped with more than 1000 ultrasonic transducers, which emit acoustic energy intracranially (i.e., to the thalamus and hypothalamus) and provide ablation and permanent disruption [71,77].

The recently described idea of combining microbubbles with their smaller derivatives (called nanodroplets) into clusters suggests that sonication-induced mechanical forces exerted by MBs on nanodroplets lead to a significant improvement in the fenestration rate and an increase in vascular permeability, confirmed by the previously adjusted extravasation into the vessel molecules [11,70]. Interestingly, in contrast with previously described data, the use of nanodroplets in glioblastoma treatment revealed a greater effect described by a higher EPR effect [78,79]. Numerous modifications of MBs’ structure have been broadly described in the literature, such as hard-shelled MBs or antibubbles with an inverted formation of the liquid and gaseous layers [80,81]. Unfortunately, their effects cannot be objectively evaluated due to the lack of standardized trials [82].

Concurrently, multiple molecules can be used as potential drug transporters, which can be easily inserted into targeted tissues. Using the HIFU method, thermosensitive structures, such as liposomes or cerasomes, provide doxorubicin release in animal cancer models [83,84].

### 2.3. Technology of FUS

The physical description of FUS does not significantly differ from that of clinical ultrasound imaging technology. While traditional ultrasound energy expands over the examined area and has recently been received and recorded as the waves echo back, FUS focuses mainly on the transfer of the acoustic energy to a previously specified location (beam focus) [77].

The main element enabling the creation of mechanical waves with a frequency of up to 7 MHz is a piezoelectric transducer with a fixed aperture and strictly assigned focal length [70,85]. The higher the frequency of the generated waves, the higher the amount of delivered energy that is later picked up by the surrounding tissues. Since temperatures lower than 55 °C are established to induce enhanced cellular permeability and facilitate the nanomolecular transport of drugs, crossing the boundary of 55 °C is connected with the induction of cell death mechanisms and coagulative necrosis [70,86,87].

## 3. Blood–Brain Barrier and Blood–Tumor Barrier Disruption

### 3.1. Structure of Blood–Brain Barrier and Blood–Tumor Barrier

Endothelial cells maintaining BBB integrity are connected with each other by tight and adherens junctions, composed of numerous proteins, like (i.a.) occludins and claudins [88]. ECs are surrounded by pericytes and astrocytic end-feet; the composition of these three kinds of cells is called neuromuscular unit (NVU) [89].

As a result of hypoxia-related increased VEGF activity, numerous processes related to tumor growth are described. Spontaneous vasculogenesis leads to augmented capillary permeability and a slightly different structure of newly created vessels. Curiously, a positive correlation between the level of expressed VEGF and MMP was noticed [90]. Due to increased MMP activity, the level of basal membrane integrity could be significantly reduced in patients with GBM [54], which predisposes tumor cells to migrate outside the vessel structure and increases the risk of metastasis [54,91]. The decreased expression of tight-junctions proteins and physical disruption of astrocytes are described as occurring simultaneously with the tumor development: BBB is disrupted, and newly created vessels surrounding the neoplastic cells create a mechanical barrier called blood–brain tumor barrier (BBTB) [14,89,92,93]. The distinct arrangement of pericites in the NVU and the reduction of astrocytic branches lead to concrete disruption and a disturbed architecture of BBB/BBTB [14,94,95]. Interestingly, connections between endothelial cells do not seem to be so dense and close that the transport of greater molecules could be disturbed [11]. Decreased levels of caveolae in irregularly composed vessels and the increased pressure of tumor tissue in the wide parenchymal area concomitantly result in an EPR effect (enhanced permeability and retention) and a lack of response during numerous anti-cancer therapies [11,96,97,98]. Dense extracellular matrix (ECM) and the attenuated lymphatic drainage of neoplastic tissues could be reasons for problems with MBs’ deposition in the tumor and halting immune cells, preventing the systemic defensive reaction [16,97,99,100,101,102,103,104,105]. Centrally located masses of the tumor seem also to be poorly supplied by blood from newly created vessels, which seem to result in limited drug deposition [14,106]. The BBB, similar to the blood–cerebrospinal fluid barrier (B-CSF-B), maintains its homeostasis due to two types of membranous transporters, responsible for the uptake of nutritious (a) and the efflux of detrimental molecules (b) [107]. A wide range of SLC family proteins are expressed in the cerebral endothelial cells and provide the transfer of ions, monocarboxylates, and amino acids, whereas ATP-binding cassette transporters (ABC-transporters)—P-glycoprotein (P-gp) and Multidrug Resistance-associated Proteins (MRPs)—guarantee the discharge of lipophilic molecules and are correlated not only with the disposal of detrimental particles but also with selective drug resistance [14,108,109]. Additionally, the general ability of transcytosis in endothelial cells in the BBB is much lower in comparison with endothelial cells located in the peripheral circulation system [14].

### 3.2. Blood–Brain Barrier Opening Mechanism

Both mechanic and ischemic injury provide an activation of numerous pathways that enable transfer via the blood–brain barrier. Whereas paracellular passage is based mainly on the tight-junction breakdown, transcellular transport is also provided by transcytosis, fenestration formation, and the direct destruction of endothelial cells [110,111,112]. Increased BBB permeability creates a route for diverse proteins to spread across endothelial cells and results in abundant vasogenic edema [33,34,35,36,37,38]. Albumins found in the parenchymal space around vessels trigger rigorous immune reactions. They not only increase microglia and trophic factor concentration but also enhance cell adhesion molecules and increase cyclooxygenase-2 expression, which can indirectly cause a significant unleash of damage-associated molecular patterns (DAMPs) [113,114,115,116,117,118,119].

As widely described in the literature, trophic factors are strictly connected with the uncontrolled opening of cationic channels in the cellular membrane, resulting in an influx of various particles (like calcium) into cells [120,121,122]. Glioma-based investigations resulted in an increased concentration of potassium channels activated by calcium, which presumably is the major cause of intensified BBB permeability [123]. Recently published reviews also underline the role of LIFUS in the management of potassium channel activity, resulting in a decrease in resting membrane potential and impeding cellular excitation, which is important when it comes to innovative drug-resistant epilepsy therapies [72,77].

Furthermore, significant increases in IL-1α, IL-1β, and TNF-α concentrations were also detected in the early phase after sonication (5–30 min) [59,118]. They lead to chemokine production and result in an increase in cell trophic factor concentration (monocyte chemoattractant protein-1 (MCP-1/CCL2)), involved in NF-KB stimulation and tight-junction disruption [59,124,125]. In vivo models suggest that the IL-1 path is strictly combined with ERK (extracellular signal-regulated kinase) phosphorylation and leads to neuronal damage, whereas TNF-α modulates AMPA/NMDA receptor subunit expression and is involved in calcium-signaling imbalance [118,126,127,128]. The delayed chemoattraction of CD68+ macrophages, observed in a few days after sonication, shows the strive of nervous cells to recover [129]. An increase in the number of both CD4+ and CD8+ subtypes of TILs (tumor-infiltrating lymphocytes) after FUS treatment was also described [130].

### 3.3. Sonication Parameters

The BBB can be differently affected depending on the sonication parameter duration, frequency of pulse repetitions (PRF), length of singular burst, and peak negative pressure index (PNP) [14]. MBs’ dosage, the size of injected molecules, encapsulated gas and bubble-shell composition, the parameters of used ultrasound, and the methods of safety control at the time of the therapy need to be discussed [131,132,133].

The main therapeutical goal is to create a structure that will not be easily disrupted in the bloodstream and that will release supplied molecules at the sonication site [11]. Since endocytosis is suspected to be the main mechanism of MB uptake in the ultrasound-stimulated area, their size becomes a crucial factor in providing effective resonance oscillation. A big-size bubble is strictly combined with decreased resonance frequency and attenuates its therapeutical properties [9,134].

The proper and effective resonance frequency of MB is determined strictly by its diameter (fR = 6.5/D) [27]. The significant change in the permeability of BBB structure was confirmed as proportionally higher only for 0.1–10 ms sonication interval since, during shorter pulses, MBs will be destroyed before they get to the main therapeutic site, although a burst length oscillating between 10 and 100 ms is believed not to cause permanent brain tissue injury [135,136]. The average time of increased endothelial permeability is estimated as 6–24 h after sonication [14,137]. The lower the size of MBs used during investigation, the weaker the impact on BBB disruption and the faster recovery to the innate state is observed [138].

To provide safe and effective sonication, MI (Mechanical Index), described as the ratio between PNP (peak negative pressure) and the square root of used ultrasound-driving frequency √f, should oscillate between 0.42 and 0.50, according to McDannold et al.’s recent study [14,139]. PNP determines the value of the oscillation amplitude generated by microbubbles, which results in the concrete type of cavitation (stable or inertial) and its consequences [140].

Animal in vitro and in vivo investigations revealed that the pressure should oscillate between 75 and 150 kPa to assess safe and effective BBB opening. The pressure level above which the stability of the environment becomes insecure is described in the literature as “the Blake threshold” and exceeding it increases the risk of inertial cavitation. Using an ultrasound center frequency near 250 kHz reduces the risk of procedure complications, maintains the effectiveness of the performed formula, and attenuates the distortion and attenuation that occur when using parameters of a higher rate. Although trials revealed that the onset of inertial cavitation for 250 kHz occurs at pressures below 200 kPa, it is worth mentioning that further investigations are required to assess the safe settlement and fit into the narrow therapeutic pressure window [141].

CI (Cavitation Index) is a ratio between negative acoustic pressure (PNP) and simple frequency without its square root. Both of them indicate the effect of cavitation on BBB structure [142,143]. The center frequency indicates the length of time of the MB exposition on the acoustic wave and the absorption of the energy thus generated [140]. As the center frequency increases, the amplitude and duration of radial oscillation decrease [140]. Nonetheless, even with similar trial protocols, their final results seem to be unpredictable due to evident personal differences in circulatory system function and its vascular structure [79].

Finding an appropriate interval between the pulses is necessary to ensure the proper circulation of MBs and their delivery to the desired region [144]. The parameter that plays the most important role in forecasting the dynamics of changes is the PRF [145]. With the increase in PL, the amount of energy absorbed by the bubbles increases, which makes it possible to lower the threshold, which, if exceeded, enables the initiation of cavitation processes. Unfortunately, too long of a PL may contribute to the premature breaking of the MBs’ structure as a result of diffusion of the gas inside [140].

A minimum off-time (measured from the formula: 1/PRF−PL = 3/volume of fluid with MBs), defining the smallest period between successive pulses enabling effective cavitation (across the 3.0 mm lateral FWHM) is (0.6/volume) seconds [140]. Furthermore, the use of a frequency lower than measured from the formula 1/minimal off-time, named as the PRF threshold, maintains a constant volume of cavitating bubbles [140]. Below this PRF threshold, modifications to pulse length did not have an impact on the SC intensity [140]. It is worth mentioning that it is necessary to use higher ultrasound intensities than in a continuous field to generate cavitation in a pulsed acoustic area.

According to the microbubbles’ structure involving a gas-filled core, they have the ability to disperse under pressure and participate in real-time imaging, which results in treating them as one of the most common ultrasound contrast agents (UCAs) [16,146]. Size up to 10 μm allows them to pass from the venous system through the heart and pulmonary vessels to the systemic circulation, which ensures their delivery through the arteries, e.g., to the nervous system [146]. Size is an important parameter when it comes to the selection of molecules involved in the intracellular transport of appropriate drugs; a few results indicate greater benefits from the use of particles of smaller diameter since, in the case of 2 µm wheals, nine-fold more surface area than the 6 µm [132].

The literature also emphasizes the need to determine the appropriate dose (volume) of MBs. The size and concentration of these small molecules can be assessed by one parameter: volume dose [132]. Interestingly, the volume of the whole solution is more important than the size of individual bubbles in the context of the effectiveness of the performed investigations [36,132]. The ability of MB molecules to remain in the bloodstream for a long time is positively correlated with the possibility of BBB penetration increase [132]. Although the size of a single MB does not affect its half-life and does not determine its longer accumulation in the bloodstream, the elongated structure of the lipid chains forming their outer layer seems to play this role [132,147,148]. Another method used to maintain MBs in the bloodstream as long as possible is their administration in the form of an extended infusion instead of several boluses [149,150].

Trials that have already been carried out revealed that a dose of 20 μL/kg enabled BBB opening, and, concomitantly with volume and dosage increase, it could enhance inflammatory response based on the CCTF and CAM increase. The lack of infectious agents co-existing with these molecules predicts the sterile inflammatory response (SIR) development [132,151].

Regardless of the content and guidelines accepted in the international protocols, MBs may cause various reactions in patients caused by different functions of, for example, the circulatory system, responsible for pumping and delivering blood with MBs to the appropriate tissues [149].

As the depth of the investigated structure increases, acoustic waves attenuate, resulting in the number of 10 cm being optimal to combine therapy effectiveness [73,152].

### 3.4. Confirmation of Blood–Brain Barrier Opening

There are multiple methods of providing BBB disruption. Histological examinations of mice-brain samples exhibited—proportionally with the concentration of used MBs—increased Evans blue (EB) staining, acting as evidence of intensive extravasation after sonication [153]. Lanthanum nitrate, accumulated in the CNS interstitial space and observed in the transmission electron microscopy (TEM), also provided an increased permeability of BBB resulting from TJ breakdown after ultrasound stimulation [153]. Decreased expression of the ZO-1, occludin, and claudin-5 proteins (elements of tight-junctions structure) was also confirmed by western blotting analysis and immunohistofluorescence [153].

Patients with high-grade glioma, treated by MRgFUS with subsequent doxorubicin/temozolomide injections, were revealed in the T1-weighted MRI areas of gadolinium enhancement, persisting even 20 h after intervention [154].

## 4. Drug Delivery

The human brain is protected by the blood–brain barrier (BBB); this structure prevents toxins, infectious agents, and drugs from entering brain cells. However, it is also a major obstacle in the treatment of brain tumors [155]. Temozolomide (TMZ) is the only chemotherapeutic agent used in chemotherapy for GBM because of its sufficient penetration across the BBB [156]. Although the effects of this treatment are poor and the median patient survival is unsatisfactory, MRgFUS, thanks to the transient opening of the BBB, makes it possible to deliver therapeutic drugs directly to the brain tissue and obtain a better effect than the actual treatment [157].

### 4.1. Enhanced Drug Carries

The MRgFUS procedure, with the intravenous injection of microbubbles, may temporarily open the BBB and BTB [156]. Several animal studies have investigated whether the opening of the BBB increases the penetration of chemotherapeutic drugs into the tumor area; these studies examined not only commonly used TMZ but also other chemotherapeutic agents, including etoposide, doxorubicin, paclitaxel, carboplatin, cisplatin, and irinotecan (Table 1). They have shown that MRgFUS increases the concentration of chemotherapeutic drugs in sonicated tumor tissue compared to non-sonicated tissue and also increases the mean brain tumor-to-serum ratio [135,158,159,160,161,162,163,164,165,166]. Additionally, reductions in tumor growth and overall survival benefits were observed in some studies in animal models [159,162,163,164]. In addition to BBB opening by FUS, Papachristodoulou et al. used liposomes with MGMT, which increased tumor cells’ susceptibility to temozolomide. The study showed its effectiveness in reducing tumor growth and survival time [167]. Until now, only one human clinical trial has been conducted; this trial enrolled six patients. Five of them underwent the full six cycles of brain–blood barrier disruption (BBBD) using FUS during TMZ therapy, and one of them underwent three cycles and then continued TMZ therapy without FUS. The TMZ therapy was started after tumor surgical resection, and patients were observed for one year after the procedure. Two of them had a recurrence of GBM after 11 and 16 months, and all of them had a survival rate of more than 1 year, which is higher than the average survival rate for GBM patients. None of the six patients experienced any complications caused by the procedure [156]. Two clinical trials using FUS-induced BBBD with TMZ chemotherapy after surgical resection are ongoing but have not published any results yet (NCT04998864, NCT03616860).

### 4.2. Drug-Loaded Microbubbles

Microbubbles are commonly used in the MRgFUS procedure; they can be used as a transfer for therapeutic drugs that can be delivered directly to the brain tissue [14]. Boron neutron capture therapy (BCNU) is a new radiotherapy method for treating malignant tumors that is characterized by selectivity and a relatively small impact on healthy cells [168]. Clinical benefits of using BCNU in GBM treatment have been shown, even though the effective delivery of the drug is essential for positive clinical outcome, but this is difficult to obtain in brain tumors [169]. Fan et al. created boron-containing nanoparticles that were conjugated to microbubbles and used combined with FUS in a mouse GBM model to increase the penetration of nanoparticles to the tumor tissue; the results showed an increase in boron uptake in the tumor area [170]. Another study on the animal GBM model used VEFG-targeted microbubbles loaded with BCNU; the results showed a significant increase in drug concentration in the targeted area and a reduction in tumor progression [171]. In addition, BCNU therapy delivered in microbubbles with the FUS procedure showed an increase in BCNU circulation time in the targeted tissue, which can reduce its toxicity [171,172]. Studies on animal models of GBM showed that using microbubbles loaded with BCNU and followed by FUS increased the survival rate and controlled tumor progression [172,173]. The main obstacle to this method is the low capacity of microbubbles; in addition, a large number of injected microbubbles are required for optimal therapeutic effect, and this needs to be tested for safety and feasibility [57].

### 4.3. Nanoparticle Delivery

Nanoparticles can act as transporters of chemotherapeutic drugs: they may increase hydrophobic drug penetration, reduce side effects, and increase the precision of administration [174]. Several studies with animal glioma models have investigated the administration of chemotherapeutic-loaded nanoparticles after MRgFUS; the drugs used in these studies were cabazitaxel, docetaxel, cisplatin, and paclitaxel [175,176,177]. Two of them reported an increase in animal survival rate after the FUS procedure and a reduction in tumor growth [176,177]. However, one using nanoparticles with capazitaxel and docetaxel showed no difference in the accumulation of the drug and therapeutic effects with and without FUS procedure [175]. One study used copper-loaded nanoparticles radiolabeled with ^64^Cu as a model drug to evaluate optimal sonication parameters, delivery efficiency, retention, and diffusion within the tumor area tested on a diffuse intrinsic pontine glioma mouse model. Four different pressures were tested: 0.28 MPa, 0.61 MPa, 0.72 MPa, and 0.85 MPa. At a pressure of 0.28 MPa, BBBD was ineffective when higher pressures significantly increased the uptake of copper in the sonicated areas. In addition, longer drug retention and more dynamic diffusion were also observed in these samples. However, a pressure of 0.61 MPa appears to be optimal because it presented similar positive effects when compared to higher pressures and also caused less hemorrhage as an adverse effect [178].

### 4.4. Immunotherapies

Another idea for the use of BBBD by FUS in glioma treatment is immunotherapy. Il-12 has been reported to have anti-angiogenic effects and promote anti-tumor immune responses [179]. An animal study examined the effect of Il-12 administration with MRgFUS causing BBBD; the results showed that an increased ratio of lymphocytes T CD8 to CD4 in the tumor region caused retarded tumor progression and an improvement in the average survival rate [180]. Another animal study used Bevacizumab, an anti-VEGF antibody that causes the inhibition of endothelial cell proliferation, which results in the inhibition of tumor neovascularization and a reduction in tumor-associated edema. The study demonstrated the improved intratumoral delivery of Bevacizumab with MRgFUS, an inhibition of tumor progression, and benefits in overall survival rate [181]. It is suspected that FUS itself causes an immune response in the area exposed to ultrasound [182]. One human–animal study verifies this hypothesis, and no immunological changes were observed in the six patients examined. In animals, an increase in the number of CD4+ and CD8+ cells was observed when using higher energy (0.81 MI), but, at lower energy (0.63 MI), this phenomenon was not observed [130].

### 4.5. Gene Therapies

Gene therapies are innovative and dynamically evolving, and ideas for their use in the treatment of glioma are emerging, even though, as with previous methods, achieving an effective concentration of therapeutic genes in tumor tissue is almost impossible due to the BBB [183]. In one study in rats, shRNA-loaded microbubbles were created and injected following FUS exposure. This shRNA had the function of inhibiting Birc5 gene transcription, which inhibits apoptosis and promotes angiogenesis. The results showed that this therapy reduced tumor growth and prolonged overall survival [184]. In another study in rats, the HSV-TK gene in the GCV paradigm was delivered into glioma in microbubbles using FUS. The HSV-TK gene is a suicide gene that promotes DNA termination, resulting in cell death; the study showed a reduction in tumor volume and an increase in median survival [185].

## 5. Sonodynamic Therapy

Sonodynamic therapy (SDT) is a modality that uses LIFUS administered in short pulses to activate sonosensitizing agents [186]. Sonoensitizers are chemical compounds activated by ultrasound stimulation that selectively accumulate in tumor cells [187]. Triggered sonosensitizers create cavitations and reactive oxygen species, inhibit angiogenesis, and induce tumor cell apoptosis [188]. Moreover, they improve the inflammatory response by activating pro-inflammatory M1 macrophages and accelerating the maturation of dendritic cells [189]. SDT has been tested in several animal studies; the most commonly used sonosensitizers are 5-Aminolevulinic acid (5-ALA) and fluorescein, which are well-known selective and safe compounds [190]. Preclinical studies using SDT in the treatment of intracranial glioma and subcutaneous glioma grafts described the inhibition of tumor expansion, decreased tumor cells viability, and increased apoptosis with no health tissue damage [191]. There are currently five ongoing clinical trials using SDT and FUS in the treatment of GBM. Two of them use 5-ALA, and three use SONALA-001 (ALA) as a sonosensitizer. One is investigating SDT with 5-ALA in newly diagnosed GBM to investigate the feasibility and safety of this therapy (NCT04845919). Three clinical trials are using SDT for recurrent GBM and are still recruiting; they aim to evaluate knowledge about safety, optimal drug doses, their toxicity, and the preparation of the optimal parameters of the FUS procedure (NCT05370508, NCT05362409, NCT04559685). One trial uses SONOALA-001 as a sonosensitizer on patients with diffuse intrinsic pontine glioma; it aims to determine the optimal and maximum tolerated dose of MRgFUS energy combined with SONOALA-001 administration.

## 6. Radiosensitization

Hypoxia in tumor tissue is believed to be the important reason for the ineffectiveness of radiotherapy in the treatment of GBM and other brain tumors that need to be overcome to increase the effectiveness of treatment [192]. FUS-induced hyperthermia may improve the effect of radiotherapy by increasing blood flow and oxygenation, resulting in reduced hypoxia [193]. Non-thermal FUS methods with microbubbles can also increase oxygen delivery to the brain tumor by creating transient gaps in cell membranes and causing the activation of immune cells [194]. Preclinical studies in mouse models with transplanted human glioblastoma cells (U87MG) have shown that radiotherapy (RT) combined with MRgFUS is more effective than RT alone, possibly due to the increased susceptibility of tumor cells DNA to damage [195]. Ying et al. showed that ultrasound-triggered microbubble destruction increases the radiosensitivity of glioma cells via the disruption of PGRMC1-mediated autophagy [196]. Another possible mechanism for increasing the effectiveness of RT combined with FUS is the reduction of the metabolic activity of glioblastoma cells and the increase in apoptosis compared to RT alone [197]. One ongoing clinical trial using NaviFUS for radiation sensitization in GBM treatment (NCT 04988750) is still recruiting and has not yet reported any results.

## 7. Histotripsy

Histotripsy is a mechanical tumor ablation using short, high-amplitude ultrasound pulses that produce bubbles that cause cavitations in the brain tissue without any thermal effects [198]. This non-thermal FUS method is more precise and has fewer side effects than thermal methods, including the thermal effect on the skin and edema around the lesion when tested on animal models [198,199]. Additionally, histotripsy enhances the anti-tumor immune response by increasing the level of immune cells and releasing anti-tumor mediators [199]. Moreover, the magnification of immune anti-tumor reaction can increase the effectiveness of checkpoint inhibition immunotherapy that is widely used in various cancers [200]. Duclos et al. tested histotripsy in murine models of primary and metastatic brain cancer using 5, 10, or 200 pulses per location at a single point of treatment or 5 or 10–20 pulses per location at multiple treatment points. The study showed that an increased number of pulses increases the hemorrhage in the targeted area and a lower number of pulses can be comparably effective to a higher number of pulses with fewer side effects [201]. Khan et al. showed on porcine models that histotripsy combined with hydrogel can increase its efficacy in degrading residual glioblastoma cells after surgical resection [202].

## 8. Tumor Ablation

Tumor ablation can be achieved by using HIFUS, causing hyperthermia in the tumor tissue; at a temperature of approximately 55 °C, neurons die as a result of protein denaturation [203]. Transcranial thermoablation has already been used to treat tremors; in this case, healthy brain cells are killed to destroy certain brain pathways that are responsible for various types of tremors [204]. The thermal ablation of the tumor is more complicated, as it is associated with a higher rate of complications, e.g., hematomas around the tumor and unwanted lesions [205]. However, some studies report the thermal ablation of GBM using FUS. One of them describes thermal ablation in 3 patients 7–10 days after craniectomy to protect skin and skull from high temperature. It shows immediate changes in imaging studies and histology indicates thermocoagulation in all three patients, even though one of them presented a neurological deficit after the procedure [206]. Coluccia et al. describe a case of a 63-year-old patient with recurrent GBM who underwent successful transcranial thermal ablation by the MRgFUS of GBM, with an improvement in neurological symptoms observed 5 days after the surgery and without any adverse events for 8 weeks of observation [207]. MacDonell et al. created the idea of an intraparenchymal catheter that allows high temperature to be delivered directly to the tumor, which can reduce side effects and increase the effectiveness of ablation. However, the implantation of the catheter is an invasive method; thus, no clinical trial has been conducted so far [208].

## 9. Liquid Biopsy

The only way to confirm the diagnosis of GBM and any other brain tumor is through the histological and molecular examination of a surgical specimen or biopsy [209]. This method is invasive and may be associated with many possible complications, including hemorrhage, infection, and postoperative neurological deficit [210]. Moreover, some patients may be disqualified due to their poor health, frailty, comorbidities, or age [57]. The precise diagnosis of the type of tumor is crucial for selecting the optimal treatment [211]. Liquid biopsy from a peripheral blood sample could detect specific tumor biomarkers in a much less invasive way [212]. Cell-free DNA (cfDNA) or short DNA fragments can be detected using the polymerase chain reaction, and then, by analyzing DNA, genetic mutations can be found and a diagnosis can be made [213]. However, brain tumors are specific due to the presence of the BBB, which prevents infection and drug toxicity, but, on the other hand, retains tumor biomarkers in the cerebral circulation [214]. MRgFUS causes a transient opening of the BBB, resulting in the release of tumor biomarkers at detectable levels into the peripheral blood circulation [215]. Table 2 presents a summary of sonication parameters in studies that examine liquid biopsy after an FUS procedure on animal models of GBM. Zhu et al. tested liquid biopsy after FUS on mouse glioblastoma models (U87 and GL261) created by the intracranial injection of enhanced green fluorescent protein (eGFP) transduced glioblastoma cells. After the FUS level of mRNA eGFP in plasma was tested by quantitative polymerase chain reaction (qPCR), the results showed that this marker was only detectable after FUS [210]. In another study on the same animal model of GBM, three different pressures of FUS sonication were compared; the study showed that the lowest pressure (0.59 MPa) caused a comparable increase in eGFP to higher pressures (1.29 MPa and 1.59 MPa). Moreover, the lowest pressure caused the lowest hemorrhage, not significantly different from the control group [216]. Pacia et al. reported an increase in myelin basic protein (MBP) and glial fibrillary acidic protein (GFAP) in plasma after the FUS procedure on porcine GBM models. In addition, no tissue damage was observed in both magnetic resonance and histological analysis [212]. Pacia et al. also presented a study in murine and porcine GBM models describing liquid biopsy after FUS, in which cell free-DNA (cfDNA) was detected. Then, mutations present in implanted cells—EGFRvIII and TERT C228T—were detected by droplet digital PCR; the results showed a significant increase in the sensitivity of mutation detection after FUS compared to liquid biopsy without FUS [217]. Meng et al. examined liquid biopsy after MRgFUS in a sample of nine patients with GBM; tested markers were cfDNA, brain-specific protein S100b, and neuron-derived extracellular vesicles (ndEV). Blood samples were collected 3 h before sonication and an average of 34 min after sonication; the results showed a significant increase in the plasma concentration of these markers [214]. Another clinical study on liquid biopsy after FUS enrolled five patients; blood samples were collected 5 min before FUS and 5 min, 10 min, and 30 min after the procedure. In the plasma of four patients, the cfDNA level was significantly higher in post-FUS samples than in the pre-FUS. In two patients, the highest level was observed after 10 min and, in two patients, after 30 min. Sonobiopsy did not show any significant damage to brain tissue [218]. One clinical trial to evaluate liquid biopsy after FUS tumor ablation is currently recruiting in Toronto (NCT04940507).

## 10. Conclusions

This review summarizes the concepts of using MRgFUS in the treatment of gliomas, especially glioblastoma multiforme. The vast majority of studies in this field are preclinical, so much time and research are still required to incorporate FUS into the treatment of gliomas. However, the results of preclinical studies are promising and indicate a positive impact on tumor progression and survival rate. Furthermore, a few side effects associated with this technique in tumor treatment are also promising. Further clinical and preclinical studies are required to determine optimal sonication parameters, including pressure, frequency, pulse duration, duty cycle, and the number of pulses; these must be thoroughly investigated to be optimal and safe in clinical practice. In addition, creating precise criteria for the tumor locations that can be treated with this procedure is extremely necessary. Establishing guidelines for the use of MRgFUS in the treatment and diagnosis of gliomas could prove to be a breakthrough in the treatment of gliomas.

## Figures and Tables

**Table 1:** Summary of recent studies regarding enhanced drug carries after FUS in animal models.

Ref.	Year	Cell Line	Drug	Tumor Growth	Survival Rate	Drug Concentration in the Brain
[158]	2021	PDGF murine glioma cells	Etoposide		No changes	Increase in drug concentration after FUS
[159]	2020	PDGF murine glioma cells	Etoposide	Decreased by 45%	Increased by 30%	Increase in drug concentration after FUS
[160]	2021	Murine model of diffuse intrinsic pontine glioma	Doxorubicin	Suppressed the volumetric tumor growth		Increase in drug concentration after FUS
[161]	2020	Mice with glioma xenografts	Paclitaxel			Increase in drug concentration after FUS
[162]	2020	Rat glioma model (F98)	Irinotecan	No changes	No changes	Increase in drug concentration after FUS
[163]	2019	Mice with U87 and PDCL glioma cells	Carboplatin	Reduced tumor growth	Prolonged survival	Increase in drug concentration after FUS
[164]	2019	Rats with F98 glioma model	Carboplatin	Reduced tumor growth	Prolonged survival	Increase in drug concentration after FUS
[165]	2018	Mice with patient-derived DIPG cells	Doxorubicin			Increase in drug concentration after FUS
[166]	2018	Mice with GBM 8401 human brain cells	Doxorubicin			Increase in drug concentration after FUS
[135]	2017	Rats with 9L gliosarcoma cells	Doxorubicin			Increase in drug concentration after FUS
[167]	2019	Mice with temozolomide-resistant gliomas	Temozolomide	Reduced tumor growth	Prolonged survival	

FUS—Focused Ultrasound Stimulation.

**Table 2:** Sonication parameters and detected biomarkers in animal studies examining FUS liquid biopsy.

Ref.	Year	Organism	Cell Line	Pressure	Ultrasound Frequency	Exposure Duration	Detected Biomarkers	Blood Sample Collection Time
[210]	2018	Mouse	U87	3.82 MPa 1.48 MPa	1.5MHz	2 min	eGFP	4 min
[210]	2018	Mouse	GL261	2.74 MPa 3.53 MPa	1.44 MHz	2 min	eGFP	
[212]	2020	Pig		1.5MPa	650 Hz	3 min	MBP GFAP	
[216]	2020	Mouse	eGFP transfected murine glioma cells	0.59 MPa 1.29 MPa 1.58 MPa	1.44 MHz	4 min	eGFP	20 min
[217]	2022	Mouse	Human GBM cells (U87)	1 MPa	1.5 MHz	3 min	cfDNA	10 min
[217]	2022	Pig	Human GBM cells (U87)	3 MPa	650 Hz	3 min	cfDNA	10 min

eGFP—enhanced green fluorescent protein; MBP—myelin basic protein; GFAP—glial fibrillary acidic protein; cfDNA—cell-free DNA.
